# Clinical Performance of the Point-of-Care cobas Liat for Detection of SARS-CoV-2 in 20 Minutes: a Multicenter Study

**DOI:** 10.1128/JCM.02811-20

**Published:** 2021-01-21

**Authors:** Glen Hansen, Jamie Marino, Zi-Xuan Wang, Kathleen G. Beavis, John Rodrigo, Kylie Labog, Lars F. Westblade, Run Jin, Nedra Love, Karen Ding, Sachin Garg, Alan Huang, Joanna Sickler, Nam K. Tran

**Affiliations:** a Department of Pathology and Laboratory Medicine, University of Minnesota, Minneapolis, Minnesota, USA; b Department of Pathology, Hennepin County Medical Center, Minneapolis, Minnesota, USA; c Department of Pathology and Laboratory Medicine, Weill Cornell Medicine, New York, New York, USA; d Department of Pathology, Anatomy, and Cell Biology, Thomas Jefferson University, Philadelphia, Pennsylvania, USA; e Department of Pathology, University of Chicago, Chicago, Illinois, USA; f Department of Pathology and Laboratory Medicine, University of California Davis, California, USA; g Roche Molecular Systems, Inc., Pleasanton, California, USA; Boston Children’s Hospital

**Keywords:** coronavirus disease 2019 (COVID-19), cobas 68/8800 SARS-CoV-2, cobas Liat SARS-CoV-2 and influenza A/B, Liat, point-of-care (POC), reverse transcription-polymerase chain reaction (RT-PCR)

## Abstract

Highly accurate testing for severe acute respiratory syndrome coronavirus 2 (SARS-CoV-2) at the point of care (POC) is an unmet diagnostic need in emergency care and time-sensitive outpatient care settings. Reverse transcription-PCR (RT-PCR) technology is the gold standard for SARS-CoV-2 diagnostics. We performed a multisite U.S. study comparing the clinical performance of the first U.S. Food and Drug Administration (FDA)-authorized POC RT-PCR for detection of SARS-CoV-2 in 20 min, the cobas Liat SARS-CoV-2 and influenza A/B nucleic acid test, to the most widely used RT-PCR laboratory test, the cobas 68/8800 SARS-CoV-2 test. Clinical nasopharyngeal swab specimens from 444 patients with 357 evaluable specimens at five U.S. clinical laboratories were enrolled from 21 September 2020 to 23 October 2020. The overall agreement between the Liat and 68/8800 systems for SARS-CoV-2 diagnostics was 98.6% (352/357). Using Liat, positive percent agreement for SARS-CoV-2 was 100% (162/162) and the negative percent agreement was 97.4% (190/195). The Liat is an RT-PCR POC test that provides highly accurate SARS-CoV-2 results in 20 min with performance equivalent to that of high-throughput laboratory molecular testing. Rapid RT-PCR testing at the POC can enable more timely infection control and individual care decisions for coronavirus disease 2019.

## Abstract (precis)

Highly accurate testing for severe acute respiratory syndrome coronavirus 2 (SARS-CoV-2) at the point of care (POC) is an unmet diagnostic need in emergency care and time-sensitive outpatient care settings. Reverse transcription-PCR (RT-PCR) technology is the gold standard for SARS-CoV-2 diagnostics.

## INTRODUCTION

A novel coronavirus, severe acute respiratory syndrome coronavirus 2 (SARS-CoV-2), the agent of coronavirus disease 2019 (COVID-19), has led to a global pandemic with widespread morbidity and mortality since first emerging in China in December 2019 (1). With limited curative therapeutics and no widely available preventative vaccination, timely and accurate diagnostic testing is vital to contain the spread of infection and reduce harmful delays in care delivery (2). However, widespread testing shortages due to the enormous global demand have hampered current diagnostic efforts (3), and longer turnaround times (TAT) of available highly accurate diagnostics can limit their clinical applications.

Consistent with recommendations from the Infectious Diseases Society of America (IDSA) (4), the majority of SARS-CoV-2 testing being performed in the United States is with nucleic acid amplification tests (NAATs), the majority of which are performed on high-throughput automated laboratory testing systems. IDSA also underscores the importance of result timeliness; however, laboratory-based platforms routinely take several hours to days to provide results and are therefore not compatible with rapid isolation of infected patients, appropriate deployment of personal protective equipment (PPE), and initiation of treatment workflows required for emergency care and time-sensitive outpatient settings.

Currently, point-of-care (POC) diagnostics for SARS-CoV-2 with TAT that are rapid (defined here as TAT of less than 30 min for all results [5]) utilize technologies (such as isothermal NAAT or lateral flow antigen testing) which are less clinically sensitive than widely used but primarily laboratory-based reverse transcription-PCRs (RT-PCRs) (6, 7). The widespread availability of sensitive and specific POC testing for SARS-CoV-2 remains an unmet diagnostic and clinical need.

In September 2020, the U.S. Food and Drug Administration (FDA) authorized the first rapid POC test for SARS-CoV-2 that utilizes RT-PCR technology (8). The cobas SARS-CoV-2 and influenza A/B nucleic acid test for use on the cobas Liat system (here referred to as Liat) (Roche Molecular Systems, Inc., Pleasanton, CA) received emergency use authorization (EUA) for the identification and differentiation of SARS-CoV-2, influenza A virus, and influenza B virus using RT-PCR in POC settings in 20 min. This multisite U.S. study is the first to evaluate the real-world performance of the Liat test for detection of SARS-CoV-2.

## MATERIALS AND METHODS

The objective of this study was to evaluate the clinical performance of the Liat for the detection of SARS-CoV-2 in nasopharyngeal swab specimens using the cobas SARS-CoV-2 nucleic acid test on the cobas 6800 and 8800 systems (here referred to as 68/8800) as the reference method.

### Liat SARS-CoV-2 and influenza A/B test.

The Liat system is for *in vitro* diagnostic (IVD) use and provides Clinical Laboratory Improvement Amendments-waived random access POC molecular testing in a compact countertop system. The system is designed to identify and measure the presence of genetic material in a biological specimen. The system automates NAAT processes, including target enrichment, inhibitor removal, nucleic acid extraction, amplification, real-time detection, and result interpretation in a rapid manner, from specimen to result in 20 min.

The Liat SARS-CoV-2 and influenza A/B test is a multiplex RT-PCR for the rapid *in vitro* detection and discrimination of RNA targets for three viruses—SARS-CoV-2, influenza A virus, and influenza B virus—in nasal or nasopharyngeal swabs preserved in transport media. In the United States, the test has been authorized for use in both laboratory and nonlaboratory POC settings (9). Test performance for influenza A/B virus detection as well as overall assay workflow and field usability were established in previous studies (10–12) of the cobas influenza A/B and respiratory syncytial virus (RSV) test (deployed in 2017 worldwide), upon which the Liat SARS-CoV-2 and influenza A/B test was built by removing RSV detection and adding SARS-CoV-2 detection. Consistent with the prospectively evaluated performance of the cobas influenza A/B and RSV test, detection of influenza A/influenza B for the cobas influenza A/B and SARS-CoV-2 test is similarly expected to yield respective values for positive percent agreement (PPAs) of 98.8 to 100% and 97.8 to 100% and values for negative percent agreement (NPAs) of 97.1 to 98.8% and 96.3 to 99.7% for influenza A and B viruses compared with other FDA-cleared and laboratory-based RT-PCRs (10, 11, 13, 14).

For SARS-CoV-2 detection, the test utilizes a dual-target design such that a positive result is generated if either or both of two target regions (ORF1a/b and N gene) of the SARS-CoV-2 genome are detected. An internal process control is added to all specimens. Limit-of-detection (LoD) studies using heat-inactivated cultured virus (USA-WA1/2020 strain, lot 324047, stock concentration of 3.16 × 10^6^ 50% tissue culture infective doses [TCID_50_]/ml; Zeptometrix, NY) demonstrated that detection rates were 95% or higher at a concentration of 0.012 TCID_50_/ml for SARS-CoV-2 (15).

### 68/8800 SARS-CoV-2 test.

The 68/8800 SARS-CoV-2 test is an analytically sensitive and accurate test with demonstrated clinical performance (16, 17); it is the most widely used platform for SARS-CoV-2 diagnostics in the United States (8, 18). The 68/8800 platform offers high-throughput testing and is automated for nucleic acid extraction and RT-PCR. Detection with 68/8800 SARS-CoV-2 utilizes a dual-target design (ORF1a/b and E gene) reported through two separate channels. Detection of either or both targets is considered a positive SARS-CoV-2 clinical result. LoD studies using heat-inactivated cultured virus (USA-WA1/2020, lot 70033175, stock concentration of 2.8 × 10^5^ TCID_50_/ml; Zeptometrix, Buffalo, NY) demonstrated that detection rates were 95% or higher for concentrations of 0.009 TCID_50_/ml and 0.003 TCID_50_/ml for target 1 (ORF1a/b) and target 2 (E gene), respectively (18). The Liat and 68/8800 tests detect different regions of ORF1a/b.

### Specimen collection and testing.

Five clinical laboratories across the United States (Davis, CA; Chicago, IL; Minneapolis, MN; New York, NY; and Philadelphia, PA) provided prospective specimens for the study. Specimens were indicated for testing based on the institutional criteria, which included the testing of both symptomatic and asymptomatic patients. Fresh and deidentified clinical nasopharyngeal swab specimens were collected into 3-ml Becton, Dickinson and Company (Franklin Lakes, NJ)/Copan (Murrieta, CA) universal transport medium (UTM) or Remel (San Diego, CA) viral transport medium (VTM) at clinical sites and tested with 68/8800 and Liat according to the EUA manufacturer instructions. While the 68/8800 test is not indicated for use with VTM according to the EUA manufacturer instructions, three clinical laboratories (CA, IL, and MN) validated VTM for routine clinical use and utilized it in the study in accordance with the study protocol. Results for SARS-CoV-2 were determined using the 68/8800 according to established testing procedures at each respective clinical laboratory; however, Liat test operators were blinded to any prior specimen test results. Each site targeted enrollment of 40 positive and 40 negative specimens, testing a mix of negative and positive specimens on each day of testing throughout enrollment. Testing was performed on both systems within 72 h of specimen collection and all specimens were stored at 2 to 8°C if testing was not performed within 4 h of specimen collection. The same specimen was used for testing on both systems.

Collected specimens were excluded for Liat testing if (i) specimen storage was not compliant with stability requirements, (ii) 500 μl of specimen was not available for Liat testing after completing testing with 68/8800, (iii) specimens were collected outside the nasopharynx, and (iv) specimens were not collected in designated media.

Liat testing was initiated on 21 September 2020 and completed on 23 October 2020. Specimen collection and testing procedures were approved by each site’s institutional review board (IRB) and were conducted in compliance with the protocol, applicable International Council for Harmonisation of Technical Requirements for Pharmaceuticals for Human Use (ICH) guidelines, and good clinical practice (GCP) guidelines. Study-specific informed consent was not necessary due to the use of remnant, deidentified specimens.

### Statistical analysis.

The overall percent agreement (OPA), PPA, and NPA for Liat results were calculated with corresponding Wilson 95% confidence intervals (CIs) compared with the positive and negative results on 68/8800. Consistent with FDA guidance, sensitivity and specificity were not used to describe the results since there is no established reference method for SARS-CoV-2 at this time. Valid and invalid test results were determined and summarized. Analyses of cycle threshold (*C_T_*) values were performed on valid test results. Reports of protocol deviations and incidents from clinical sites were evaluated descriptively. All data analyses were performed using SAS/STAT software (version 9.4; SAS, Cary, NC). Based on *a priori* power and statistical calculations, collection of at least 120 positives (as determined by 68/8800) was targeted in order to achieve PPA/NPA of approximately 98% and a 95% lower CI bound of approximately 95%. *C_T_* values were recorded for both tests to support investigation of discordant results.

## RESULTS

### Specimen disposition.

A total of 444 nasopharyngeal swab specimens were collected from five study sites. Eighty-four specimens (32 positives and 52 negative) did not meet the inclusion criteria and were ineligible: 51 due to the utilization of off-protocol collection media, 28 due to testing outside the stability window, and 5 due to pooled 68/8800 testing. Three specimens were nonevaluable since they were invalid upon initial and repeat Liat testing. There were 357 evaluable specimens (162 SARS-CoV-2-positive and 195 SARS-CoV-2-negative specimens) based on the result from the 68/8800 test included in the final analysis. Enrollment and representation of positive specimens were not equally distributed across study sites due to differences in COVID-19 prevalence and availability of specimens collected in eligible collection media. As a result, two sites (CA and MN) were asked to extend enrollment beyond 40 positive and 40 negative specimens, because they were not impacted by these limitations. Specimen disposition is shown in Table 1.

**TABLE 1:** Specimen disposition of the 444 nasopharyngeal swab specimens collected and tested in the study^*a*^

Site	Eligible					Noneligible and excluded due to protocol deviation				Total
	Evaluable			Nonevaluable on Liat and excluded		*n*	Off-protocol transport media	cobas 68/8800 testing in pooled specimens	Out of 72-h window	
	*n*	+	−	*n*	Invalid Liat result					
CA	105	52	53	0	0	1	0	0	1	106
IL	34	16	18	1	1	27	0	0	27	62
MN	116	58	58	1	1	0	0	0	0	117
NY	34	18	16	1	1	56	51	5	0	91
PA	68	18	50	0	0	0	0	0	0	68
Total	357	162	195	3	3	84	51	5	28	444

a Abbreviations: CA, University of California, Davis, CA; IL, The University of Chicago Medicine, Chicago, IL; MN, Hennepin County Medical Center, Minneapolis, MN; NY, New York-Presbyterian Hospital/Weill Cornell Medical Center, New York, NY; PA, Jefferson Hospital, Philadelphia, PA. +, positive; −, negative.

### Test performance.

Table 2 compares the performance between the Liat and 68/8800 tests. The Liat test demonstrated 100% PPA (95% CI, 97.7 to 100%), 97.4% NPA (95% CI, 94.1 to 98.9%) and 98.6% OPA (95% CI, 96.8 to 99.4%) with the 68/8800. In total, 354 specimens yielded initial valid results on the Liat and 6 specimens yielded an initial invalid result (initial invalid rate, 1.66% [6/360]), out of which 3 yielded valid results when repeated per the instructions for use; this resulted in the 357 evaluable specimens.

**TABLE 2:** Comparison of Liat with 68/8800 for the detection of SARS-CoV-2

cobas Liat SARS-CoV-2 result	No. of samples with indicated cobas 68/8800 SARS-CoV-2 result		Total
	Positive	Negative	
Detected	162	5	167
Not detected	0	190	190
Total	162	195	357

### Discordant specimens.

**FIG 1:**
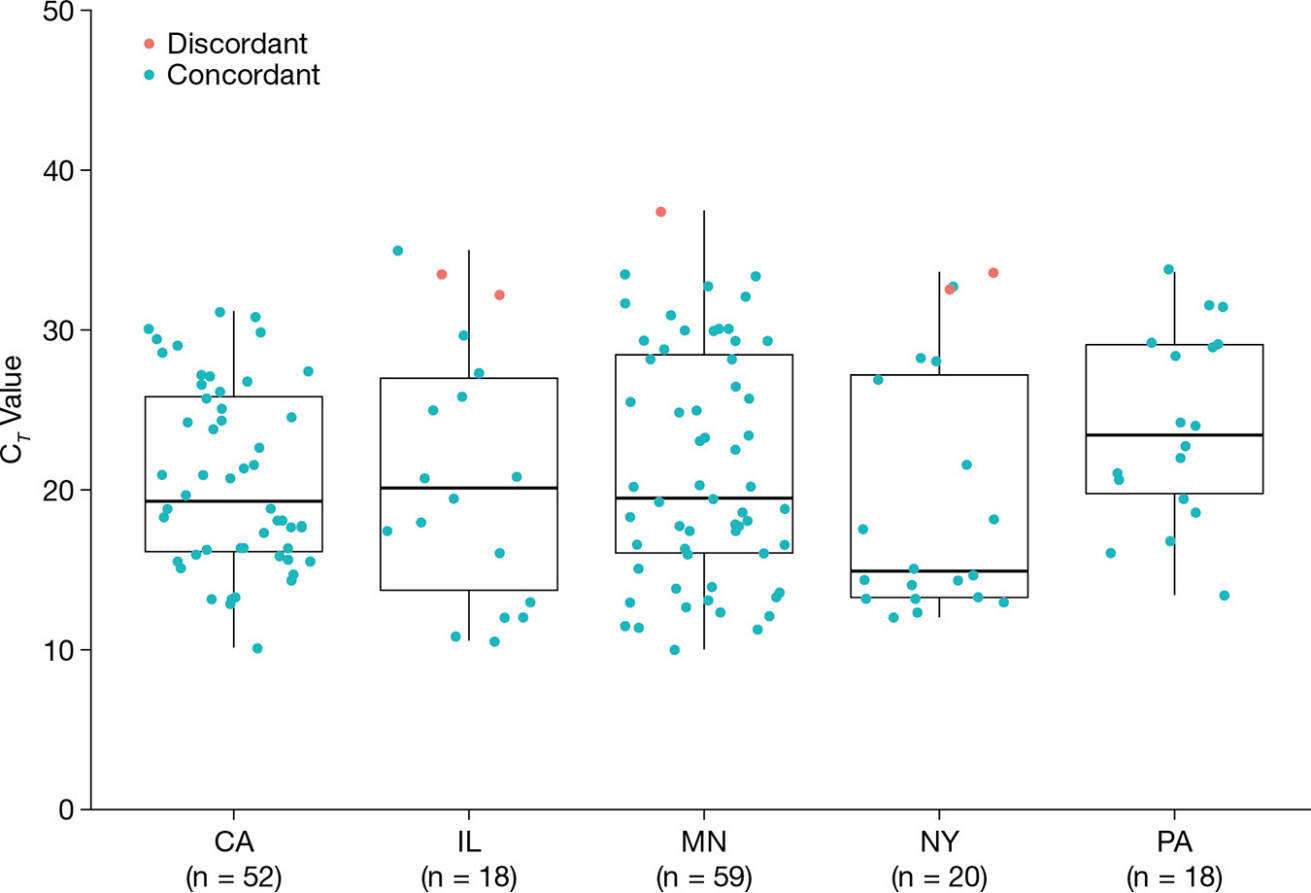
*C_T_* values for all evaluable Liat-positive results by site. All 167 evaluable specimens with positive Liat results were included, including the 5 discordant specimens.

Five specimens were discordant, with Liat-positive and 68/8800-negative results: four specimens were collected in UTM that is indicated for use by both tests and one specimen was collected in VTM; this was on protocol but is indicated for use with the Liat only. Figure 1 shows the *C_T_* values for all Liat-positive results. Discordant specimens all had relatively delayed *C_T_* values (indicative of a lower viral concentration in the specimen) of 32.2, 32.6, 33.5, 33.6, and 37.4. Concordant specimens had *C_T_* values ranging from 10.1 to 35.0, with median of 19.5.

## DISCUSSION

The Liat test demonstrated excellent test agreement (98.6% OPA, 100% PPA, and 97.4% NPA) with a widely used laboratory RT-PCR for SARS-CoV-2 detection in a multisite U.S. study. With a 20-min TAT and EUA approval for POC use, this study demonstrates that the Liat test enables rapid and accurate POC detection of SARS-CoV-2.

Discordance between comparably performing molecular tests—in this case the Liat and 68/8800 tests—is commonly observed for SARS-CoV-2 and other microorganisms when very low concentrations of viral RNA are present in the specimen (16, 17). In this study, all five discordant specimens had visually delayed *C_T_* values (Fig. 1) compared to the concordant specimens, indicative of lower viral concentrations. Moreover, these discordant specimens had *C_T_* values near the LoD previously reported for the Liat test (19). LoD evaluations suggest that comparable and very low viral concentrations can be detected by both assays; however, the translation of analytical to clinical sensitivity can be impacted by many variables, such as testing conditions and normal run-to-run variation. Ultimately, the clinical significance (such as transmissibility and infectiousness) for patients with specimens containing low concentrations of SARS-CoV-2 is not fully understood and is under active investigation (20).

Several current POC tests using non-RT-PCR technologies for SARS-CoV-2 can be rapidly performed, i.e., in less than 30 min; however, poorer analytical sensitivities (relative to RT-PCR) of these tests translate to increased clinical false negativity (21–23). For example, following EUA availability of the POC Abbott ID NOW COVID-19 test, widespread adverse-event reporting of inaccurate negative results led to a recommendation from the FDA to confirm all negative SARS-CoV-2 results with a sensitive molecular test (24). This finding for SARS-CoV-2 testing is consistent with the performance of similar non-RT-PCR POC tests for influenza, where rapid diagnostic tests, diffusion immunoassays, and isothermal POC NAATs have documented lower performance than RT-PCR (25).

The unmet and enormous global demand for SARS-CoV-2 tests has also resulted in significant public discourse regarding the benefits of highly accurate and clinically sensitive NAATs given the existing constraints (availability and ease of use) compared with lower-clinical-sensitivity technologies, like rapid antigen tests, that are easier to deploy at high volumes (26, 27). In outpatient settings where the alternative choices are either “no test” or an accurate laboratory-based molecular test (with a long TAT), the benefits of less sensitive rapid antigen technologies should be considered. However, in other contexts, such as where critical infection control decisions rely on accurate test results, rapid assays with lower clinical sensitivity are not acceptable. Future research is required to validate these various testing modalities and define strategies that optimize clinical performance and reagent supply as the pandemic evolves.

In contrast to rapid tests using non-RT-PCR technologies, the Liat test provides speed and highly accurate and clinically sensitive performance (21). To date, the only other RT-PCRs for SARS-CoV-2 close to this profile are the Xpert Xpress SARS-CoV-2 (28) and Xpert Xpress SARS-CoV-2/Flu/RSV assays (29) when used at the POC on the Xpert Xpress system, with TAT of 45 min (30-min early callout for positives) and 36 min (25-min early callout for positives), respectively. Rapid highly accurate specimen-to-answer platforms are not scalable to replace high-throughput automated laboratory systems. Instead, they should be deployed to complement these platforms. The Liat can add significant value in settings where immediate accurate results for individual patients can improve care and reduce the use of valuable resources. Examples include emergency settings, time-sensitive procedures such as surgery, and targeted outpatient settings.

In emergency settings, it is crucial to know whether an individual patient is positive for the virus. During peak periods of the pandemic, effective use of resources is critical, both to improve morbidity and mortality of SARS-CoV-2 patients and to prevent the further spread of the virus. Identifying SARS-CoV-2-positive patients at the point of admission facilitates this by enabling effective patient cohorting and discharge to congregate living facilities, providing information about the level of infection control required, targeting resources such as PPE appropriately, and accelerating enrollment into drug trials and/or administration of EUA therapies.

For time-sensitive procedures and high-risk surgeries, especially continuous-aerosol-generating procedures (such as in orthopedics [30] and otorhinolaryngology [31]), many hospitals are using a COVID-19 testing protocol which can be instituted up to 96 h in advance of hospital admission to confirm that patients are negative (32). Preprocedure testing often relies on high-throughput laboratory-based platforms and has limited use for urgent or unexpected problems encountered in trauma, emergency medicine, deceased-donor transplant (33), and obstetrics. For these special cases, rapid confirmation of negative SARS-CoV-2 status saves time and resources by preventing unnecessary use of contact precautions.

Outpatient settings may require confirmation of negative SARS-CoV-2 status for the patient to receive care. The Liat SARS-CoV-2 influenza A/B test provides accurate rapid-response testing for symptomatic individuals with preexisting risk factors for COVID-19 mortality/morbidity. The fast TAT (20 min) of the Liat could also be leveraged by health care facilities for symptomatic frontline employees and COVID-19 cluster investigation to quickly rule out negative individuals and facilitate their return to work.

The Liat SARS-CoV-2 and influenza A/B test that was utilized in this evaluation has the additional benefit of testing for influenza at the same time as SARS-CoV-2; this provides further important information for symptomatic patients by allowing differential diagnosis when multiple respiratory viruses are in circulation (34). POC RT-PCR testing for influenza in the clinic can be used to guide patient care decisions (35) and has been shown to be effective at reducing inappropriate prescribing of antibiotics (36) as well as potentially reducing the length of stay in hospital (37). In addition, the Liat has demonstrated cost savings to hospital systems when deployed in the emergency department for respiratory pathogen testing (34, 35).

This study has several limitations. First, clinical patient-level data (such as the duration of symptoms) were not collected and analysis was limited to test performance comparisons; this limited further investigation into the clinical significance of specimens with very low concentrations of virus. Second, because the enrollment period occurred prior to the start of the winter respiratory season, we were not able to assess the clinical performance for influenza A and B detection in this study. Third, an equal proportion of negative specimens was included in the study in order to preserve scarce testing supplies for clinical use, so prevalence does not reflect a real-world population. Lastly, further testing of the specimens to investigate discordant results was not possible.

In conclusion, the Liat is the first RT-PCR assay capable of reporting results in 20 min at the point of care. The evaluation demonstrates equivalent performance to high-throughput laboratory RT-PCR testing and fills an unmet diagnostic need in frontline settings to accurately and rapidly test for SARS-CoV-2. This favors use in settings where immediate decision making is required for treatment, PPE, and infection prevention decisions.
